# Preemptive nalbuphine combined with subcostal transversus abdominis plane block for multimodal analgesia in laparoscopic liver resection: a randomized controlled trial of multimodal analgesia for laparoscopic hepatectomy

**DOI:** 10.1016/j.clinsp.2026.101081

**Published:** 2026-09-05

**Authors:** Fang He, Peng Jiang, Dexing Luo

**Affiliations:** Department of Anesthesia, Huizhou Central People's Hospital, Huizhou City, Guangdong Province, China

**Keywords:** Laparoscopic liver resection, Nalbuphine, Preemptive analgesia, Subcostal transversus abdominis plane block

## Abstract

**Background:**

Postoperative pain control is crucial for Laparoscopic Liver Resection (LLR) patients, as poor analgesia may hinder recovery and elevate complication risks.

**Methods:**

180 LLR patients were randomized into three groups (n = 60 group): Control (C), subcostal TAPB alone (TAPB), and Nalbuphine + subcostal TAPB (N+TAPB). Outcomes included NRS, BCS, Hemodynamics (MAP, HR), PCIA metrics, QoR-15 scores and complications.

**Results:**

Across the postoperative time points, the NRS scores in Group N+TAPB were significantly lower than those in Group C and Group TAPB (p < 0.05), whereas BCS scores were significantly higher (p < 0.05). At the time points of endotracheal intubation, skin incision, and immediately post-extubation, both MAP and HR in Groups N+TAPB and TAPB were significantly lower than those in Group C (p < 0.05). At 24 h after surgery, the effective number of PCIA compressions and the total dose of rescue non-steroidal anti-inflammatory drugs (parecoxib sodium and/or flurbiprofen axetil, in mg) were significantly lower in Group N+TAPB than in Group C and Group TAPB (p < 0.05). Moreover, the QoR-15 score at 24 h after surgery was highest in Group N+TAPB (p < 0.05). Regarding safety and tolerability, the incidences of nausea, vomiting, and pruritus in Group N+TAPB were significantly lower than those in Group C (p < 0.05).

**Conclusion:**

The regimen combining preemptive nalbuphine with subcostal TAPB was associated with better postoperative analgesia, more stable perioperative hemodynamics, and fewer opioid-related adverse events than no regional block and represents a feasible multimodal analgesia strategy for LLR. Because the control group did not receive a sham TAPB, the specific pharmacological contribution of TAPB alone cannot be isolated from a procedural placebo effect.

## Highlights

• Comparison of baseline characteristics between the three groups of patients.
• Comparison of analgesic effects among the three groups of patients.
• Comparison of hemodynamic indexes among the study groups.
• Comparison of postoperative analgesia indicators among the three groups of patients.
• Comparison of postoperative recovery quality and complications among three groups of patients.

## Introduction

Laparoscopic Liver Resection (LLR), as a landmark technique in the evolution of minimally invasive hepatobiliary surgery, has been widely used in the treatment of benign and malignant liver lesions because it is associated with less surgical trauma, reduced intraoperative bleeding, and faster postoperative recovery.^1^^,^^2^ However, postoperative pain after LLR remains a central barrier to early rehabilitation. The sources of pain are multifactorial, including somatic pain related to trocar placement and abdominal wall incisions, visceral pain caused by traction of the liver capsule and intra-abdominal visceral stimulation, and referred or compression-related pain resulting from pneumoperitoneum pressure on the diaphragm.^3^^,^^4^ When postoperative analgesia is inadequate, patients may develop restricted breathing and delayed mobilization, and the risk of complications such as pulmonary infection and deep vein thrombosis may increase, which is inconsistent with the principles of Enhanced Recovery After Surgery (ERAS).^5^ Accordingly, multimodal analgesia, by combining analgesic approaches with different mechanisms of action to achieve a synergistic “1 + 1 > 2″ effect, has become the current mainstream strategy for postoperative pain management.^6^

Nalbuphine, a novel opioid receptor agonist-antagonist, exerts strong agonistic activity at κ-receptors and partial activity at μ-receptors, thereby offering distinctive potential advantages for postoperative analgesia.^7^^,^^8^ κ-receptor is widely distributed in the spinal cord and peripheral levels, and its activation can effectively relieve visceral pain and deep somatic pain by inhibiting the excitability of dorsal horn neurons and reducing the release of nociceptive transmitters such as substance P. Compared with a μ receptor agonist alone, nalbuphine provides equivalent analgesia and has a lower incidence of adverse reactions such as respiratory depression, nausea and vomiting. Subcostal Transversus Abdominis Plane Block (TAPB) represents an important development in regional anesthesia techniques because, through injection of local anesthetic into the plane between the transversus abdominis and rectus abdominis muscles, it blocks the anterior and lateral cutaneous branches of the T7–T12 intercostal nerves and provides targeted analgesia for the anterior abdominal wall, which is particularly applicable to pain control after upper abdominal surgery.^9^^,^^10^ However, a single analgesic regimen has inherent limitations. Previous studies have indicated that nalbuphine alone may require dose escalation to cover both visceral and somatic pain, with a potential risk of excessive sedation^11^ conversely, although subcostal TAPB alone can alleviate abdominal wall pain, it is often insufficient to effectively suppress visceral pain and pneumoperitoneum -related pain.^12^ This study aims to investigate the combined use of nalbuphine preemptive analgesia and subcostal TAPB to clarify the effectiveness and safety of this strategy, to provide a more favorable clinical option for postoperative analgesia in LLR, and to facilitate deeper implementation of the ERAS concept in hepatobiliary surgery.

## Data and methods

### Ethics

This study was approved by the ethics committee of the hospital, all patients signed informed consent forms, and the study was registered in ISRCTN (registration number: ISRCTN18315487) at https://www.isrctn.com/ISRCTN18315487. The study was conducted in accordance with the Declaration of Helsinki. This randomized controlled trial followed the CONSORT Statement guidelines for reporting.

#### Study participants

A total of 180 patients scheduled for laparoscopic liver resection at Huizhou Central People’s Hospital between September 2024 and September 2025 were prospectively enrolled. The sample size was calculated as follows: based on previous experiments and a related literature review,^13^ the incidence of moderate-to-severe pain in the blank control group was 50%, whereas the incidence in the other groups was 25%–35%; therefore, the sample size was calculated using an expected minimum between-group difference of 25%. With the power set at 80% and the significance level set at 0.05, the calculation indicated that at least 55 patients were required per group. Considering a 10% loss to follow-up or dropout rate, 60 patients were included in each group, resulting in a total sample size of 180 participants. As a sensitivity check on the primary continuous outcome, assuming a clinically meaningful difference of 1.5 points on the postoperative NRS at 24 h (estimated SD=2.0) between groups, a sample of 50 patients per group would also provide approximately 80% power to detect such a difference at a two-sided α=0.05; the 60 patients per group enrolled in the present study therefore provided adequate power for both the binary (moderate-to-severe pain incidence) and continuous (NRS) framings of the primary outcome.

Inclusion criteria: a) American Society of Anesthesiologists (ASA) grade I–III, age 18–70 years, Body Mass Index (BMI) 20–25 kg/m^2^. b) Preoperative laboratory tests, including liver and kidney function and coagulation function, were basically normal. c) The abdominal skin was intact, without damage or infection.

Exclusion criteria: a) Cardiovascular or cerebrovascular diseases, or dysfunction of important organs such as the liver or kidney. b) Abnormal coagulation function or bleeding tendency. c) History of chronic pain or long-term use of opioid analgesics; patients with autoimmune diseases or immune dysfunction. d) Mental system disorders or cognitive impairment; pregnant or lactating women.

Test termination criteria: a) Conversion to laparotomy during the operation. b) Concomitant performance of other operations. c) Patients voluntarily requested withdrawal during the trial. d) Serious adverse events or transfer to the ICU during the trial; severe postoperative complications (infection, etc.).

**Fig. 1:**
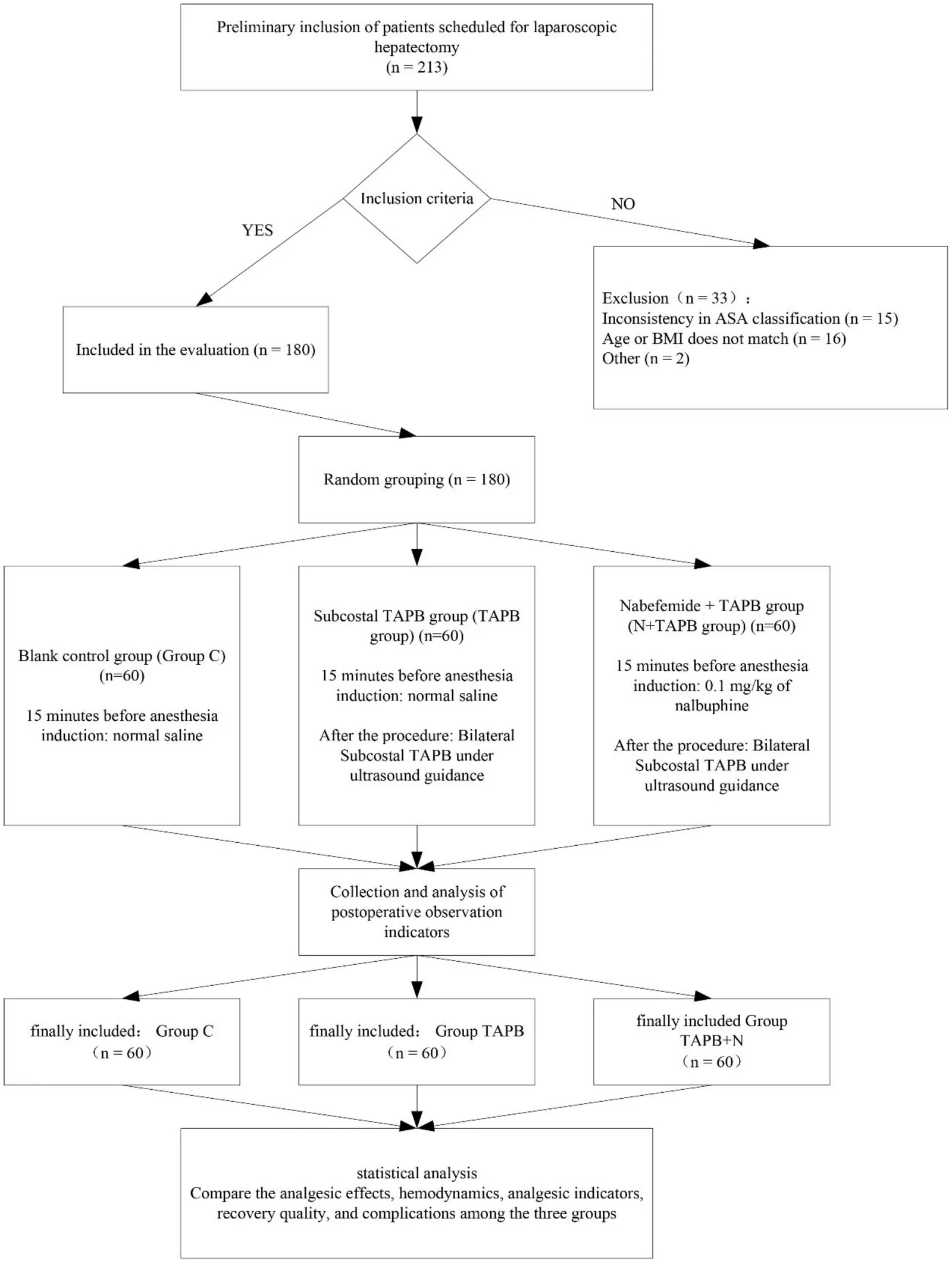
Inclusion process for study enrollment.

Randomization and blinding: The participants were randomly assigned using a computer-generated random number sequence, with allocation concealed via sealed opaque envelopes. Due to the nature of the interventions, patients and anesthesiologists were not blinded; however, outcome assessors (collecting NRS, BCS, QoR-15) were blinded to group assignment. Patients were divided into three groups: control group (C group), a subcostal approach TAPB group (TAPB group), and a nalbuphine + subcostal TAPB group (N+TAPB group) using a random number table method, with 60 cases in each group. All surgeries were performed by the same group of physicians. The inclusion process of study participants is shown in Fig. 1.

### Anesthesia

Preoperative visits and patient education were conducted routinely, during which the 15-item Quality of Recovery Scale (QoR-15) and the Numerical Rating Scale (NRS) were explained in detail to ensure patient understanding and cooperation. After the patient entered the operating room, electrocardiography, noninvasive blood pressure, Heart Rate (HR), peripheral oxygen saturation, and body temperature were continuously monitored, while peripheral venous access was established, and radial artery catheterization was performed for invasive arterial pressure monitoring. The depth of anesthesia was monitored using the Narcotrend® system.

Anesthesia was induced with intravenous midazolam (0.05 mg/kg) in combination with Target-Controlled Infusion (TCI) of propofol (initial effect-site concentration: 4 μg/mL) and remifentanil (initial effect-site concentration: 3 ng/mL). After loss of consciousness and when the Narcotrend index decreased to below 50, cisatracurium (0.15 mg/kg) was administered intravenously, and tracheal intubation was performed after 3-min of assisted ventilation. Following successful intubation, mechanical ventilation was initiated and respiratory parameters were adjusted according to intraoperative needs. Anesthesia was maintained using sevoflurane inhalation together with TCI of propofol and remifentanil, while the Narcotrend index was controlled within D1–E0 based on EEG monitoring; additionally, cisatracurium (0.1 mg/kg) was administered as required according to the clinical signs of neuromuscular recovery and fading muscle relaxation.

After surgery, the patient was transferred to the post-anesthesia care unit, and the tracheal tube was removed once consciousness and spontaneous breathing were adequately restored. If intraoperative blood pressure or HR increased or decreased to 20% of the baseline values measured at home, vasoactive agents were administered to restore these parameters to an appropriate range. A Patient-Controlled Intravenous Analgesia (PCIA) pump was initiated at the end of surgery, with the analgesic solution consisting of hydromorphone (6 mg) and tropisetron hydrochloride (15 mg) diluted to a total volume of 150 mL. If the NRS score was >4-points after operation, paracetamol (1 g), parecoxib sodium (40 mg) or flurbiprofen axetil (50 mg) was given intravenously as rescue analgesia according to the patient's situation. The choice of drugs was determined by the attending physician in the Department of Anesthesiology according to the patient's liver and kidney function and allergic history. The three drugs are commonly used non opioid analgesics in clinical practice. Parecoxib sodium and flurbiprofen axetil are both non-steroidal anti-inflammatory drugs, and their doses were combined into “total non-steroidal anti-inflammatory drugs (mg)” in the comparison between groups; Acetaminophen is recorded as a separate category. Parecoxib sodium 40 mg and flurbiprofen axetil 50 mg were within the clinical equivalent dose range, without weighted conversion, and were directly accumulated in the original milligrams. C group: The patients received physiological saline 0.1 mL/kg 15-min before anesthesia induction, and anesthesia was then managed according to the standard protocol.

TAPB group: The patients received physiological saline 0.1 mL/kg 15-min before anesthesia induction, followed by anesthesia according to the standard protocol; upon completion of surgery, bilateral subcostal TAPB was performed under ultrasound guidance.

N+TAPB group: The patients received intravenous nalbuphine (0.1 mg/kg) 15 min before anesthesia induction, after which anesthesia was managed according to the standard protocol; upon completion of surgery, bilateral subcostal TAPB was performed under ultrasound guidance.

Subcostal TAPB technique: The ultrasound probe was first placed parallel to the xiphoid process to visualize the linea alba and the bilateral rectus abdominis muscles, after which the probe was rotated to align parallel to the costal margin and then moved obliquely outward. In this plane, the transversus abdominis plane lies between the rectus abdominis and transversus abdominis muscles, and the TAP was identified along the oblique line extending from the xiphoid process toward the anterior superior iliac spine under the costal margin. An in-plane needle approach was used, and hydrodissection was performed to confirm correct placement; after negative aspiration, 20 mL of 0.375% ropivacaine was injected. The dosage of narcotic drugs was determined based on clinical experience and previous studies. Nalbuphine 0.1 mg/kg is the initial dose commonly used for postoperative analgesia. As for the timing of administration, the peak time of plasma concentration after intravenous injection of nalbuphine is about 2∼5 min, and the effective analgesic concentration can last for about 30∼60 min. In this study, the drug was administered 15-min before anesthesia induction to ensure that the drug had been fully distributed to the central action site before the occurrence of strong noxious stimuli such as endotracheal intubation and surgical skin incision, so as to realize the drug coverage of preemptive analgesia.

Intraoperative opioid administration was standardized across all groups. Remifentanil was administered using target-controlled infusion with an effect-site concentration of 2–4 ng/mL, and adjustments were made only when MAP or HR deviated more than ±20% from baseline. Depth of anesthesia was maintained within the Narcotrend range D1–E0. No additional long-acting opioids were administered intraoperatively. This protocol ensured minimization and consistency of opioid exposure among groups.

### Observation indicators

#### Baseline characteristics and surgical parameters

Recorded data included age, gender, Body Mass Index (BMI), American Society of Anesthesiologists (ASA) physical status classification, duration of surgery, duration of anesthesia, hepatic portal occlusion time (Pringle maneuver time), intraoperative blood loss, and extubation time.

#### Main indicators

The primary outcome was the NRS score at 24 h postoperatively. NRS, Bruggemann Comfort Scale (BCS), and Ramsay scores were recorded at 2, 8, 12, and 24 h after the operation. The NRS is a simple and intuitive pain assessment tool that quantifies patients’ subjective pain intensity on a scale from 0 to 10, with higher scores indicating more severe pain.^14^ BCS was used to evaluate pain-related comfort on a scale from 0 to 4, with higher scores indicating greater comfort.^15^ The Ramsay sedation score, a classic instrument for evaluating sedation depth, was used. It ranges from 1 to 6, with higher scores reflecting deeper sedation; a postoperative score between 2 and 3 is generally considered to represent satisfactory sedation.^16^ NRS (0–10), BCS (0–4), and Ramsay (1–6) scores were assessed by trained, blinded staff using validated Chinese versions of the scales.

#### Hemodynamic indicators

The depth of anesthesia was continuously monitored, and hemodynamic parameters were collected at four key time points: before anesthesia induction, immediately after tracheal intubation, immediately after skin incision, and immediately after tracheal extubation. Mean Arterial Pressure (MAP) was recorded at each corresponding time point, while heart rate (HR) was obtained from continuous ECG monitoring at the same time points.

#### Postoperative analgesia indicators

For patients in the three groups, the effective number of compressions of the Patient-Controlled Intravenous Analgesia pump (PCIA) within 24 h after surgery was recorded. In addition, the total dosage (mg) of parecoxib sodium or flurbiprofen axetil administered within 24 h postoperatively was recorded for each group.

#### Postoperative recovery quality

The incidence of adverse reactions (including nausea, vomiting, respiratory depression, and other symptoms) was compared among the three groups. Postoperative recovery quality was also compared across groups, and the QoR-15 score at 24 h after surgery was recorded. The QoR-15 comprises 15-items across five dimensions ‒ physical independence, pain, physical comfort, psychological support, and emotional state ‒ and yields a total score ranging from 0 to 150, with higher scores indicating better recovery quality.^17^

### Statistical analysis

Data analyses were performed using SPSS 26.0 software. Measurement data are presented as mean ± standard deviation ($\bar{x}$±s). For comparisons among multiple groups, one-way analysis of variance (ANOVA) is employed. For data on primary outcome measures (NRS, BCS) that change over time, two-way repeated-measures ANOVA is used, with treatment group and time as the main effects, and the interaction between group and time is tested. When the interaction is statistically significant, one-way ANOVA is further conducted at each time point, followed by Bonferroni correction for post-hoc multiple comparisons to control the overall Type I error rate. For other non-repeated-measures measurement data, one-way ANOVA is used for comparisons among multiple groups, with Bonferroni correction applied for post-hoc comparisons. For all analyses involving multiple comparisons, the Bonferroni method is utilized to adjust the significance level and control the family-wise error rate. Categorical data were expressed as rates (%), and differences between groups were analyzed using the χ^2^ test. Ordinal data (Ramsay score) were analyzed using the Kruskal-Wallis test. A two-sided p-value of <0.05 was considered statistically significant.

## Results

### Comparison of baseline characteristics between the three groups of patients

No significant differences were observed among the three groups with respect to baseline characteristics, including age, sex, BMI, ASA classification, surgical time, anesthesia time, hepatic portal occlusion time, intraoperative blood loss, extubation time, and intraoperative anesthetic drug use (all p > 0.05), indicating that the three groups were comparable at baseline (Table 1).

**Table 1:** Comparison of baseline characteristics of three groups of patients.

Index	C group (n = 60)	TAPB group (n = 60)	N+TAPB group (n = 60)	Statistical value	p-value
Age (years, $\bar{x}$±s)	49.03±9.43	48.78±9.99	49.35±9.03	F = 0.054	0.948
Gender (male/female, example)	32/28	30/30	31/29	χ^2^ = 0.133	0.935
BMI (kg/m^2^, $\bar{x}$±s)	22.61±1.41	22.15±1.48	22.59±1.56	F = 1.793	0.169
ASA grading (I/II/III, example)	15/38/7	14/39/7	16/37/7	χ^2^ = 0.186	0.996
Surgical time (min, $\bar{x}$±s)	133.67±19.27	134.34±21.37	133.12±22.06	F = 0.051	0.950
Anesthesia time (min, $\bar{x}$±s)	169.05±19.71	160.14±22.42	166.39±22.22	F = 2.720	0.069
Hepatic portal block time (min, $\bar{x}$±s)	35.98±6.29	35.08±6.39	34.72±5.75	F = 0.674	0.511
Bleeding volume (mL, $\bar{x}$±s)	125.71±36.64	115.97±34.94	121.28±37.05	F = 1.130	0.325
Extubation time (min, $\bar{x}$±s)	15.16 ± 3.98	14.65 ± 4.09	13.93±3.62	F = 1.512	0.223
Intraoperative anesthetic drug dose					
Sufentanil (μg, $\bar{x}$±s)	28.52±4.36	27.98±4.15	28.16±4.52	F = 0.213	0.808
Remifentanil (μg/kg, $\bar{x}$±s)	12.35±2.18	11.96±2.34	12.12±2.27	F = 0.457	0.634
Propofol (mg/kg, $\bar{x}$±s)	4.82±0.75	4.76±0.81	4.85±0.79	F = 0.189	0.827
Vasoactive drug use, n (%)	8 (13.33%)	7 (11.67%)	6 (10.00%)	χ^2^ = 0.452	0.798

### Comparison of analgesic effects among the three groups of patients

**Fig. 2:**
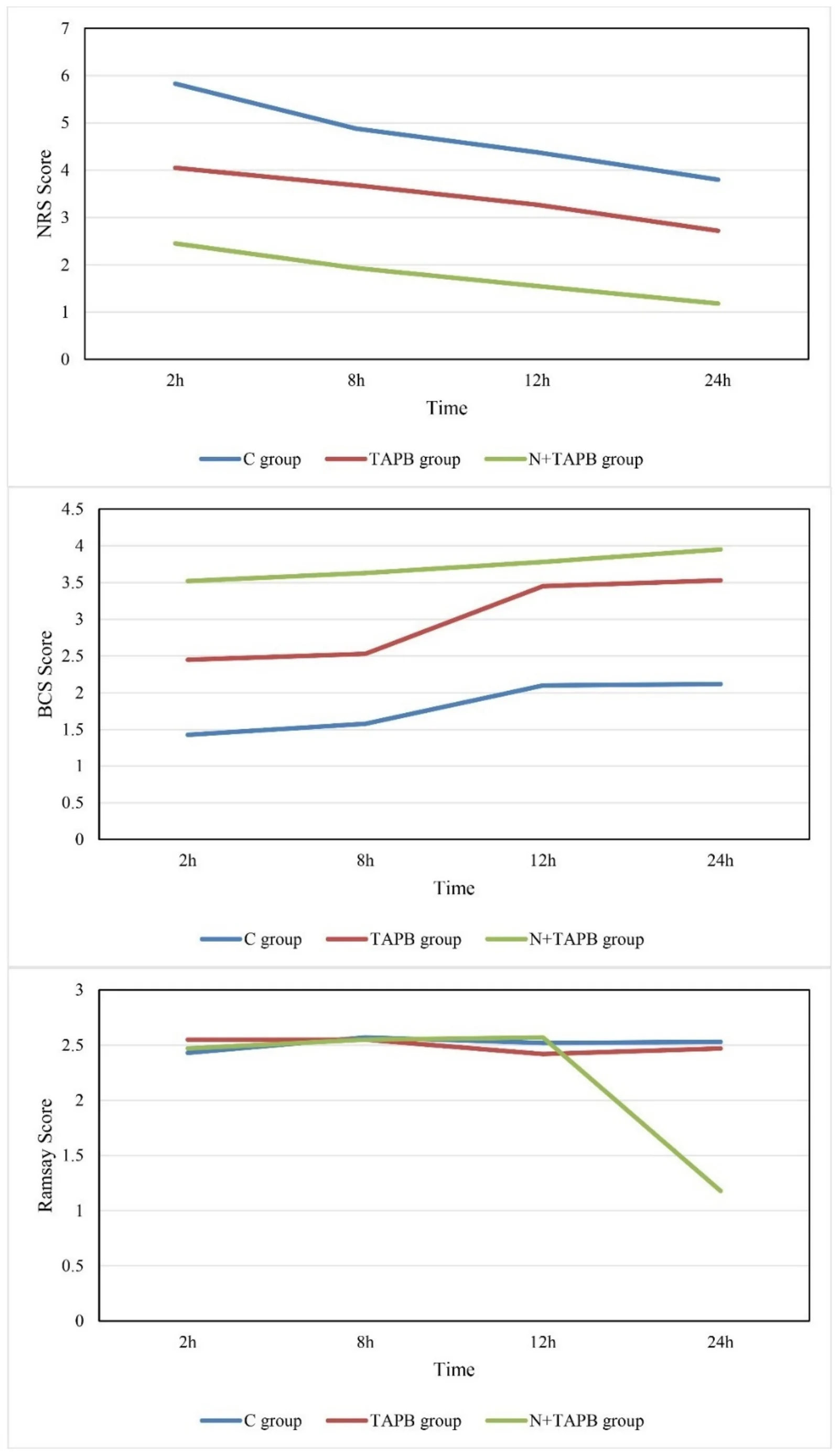
Postoperative NRS scores, BCS scores, and Ramsay scores for three groups of patients. Error bars represent Standard Deviation (SD). Note: The figure clearly shows the trend of postoperative NRS scores, BCS scores, and Ramsay scores over time in the three groups. The lower the NRS scores, Ramsay scores, and the higher the BCS scores, the better the analgesic effect.

The results of two-factor repeated-measurement ANOVA showed that group (F = 42.36, p < 0.001), time (F = 18.72, p < 0.001) and the interaction between group and time (F = 6.85, p < 0.001) had significant effects on NRS score. BCS score also showed similar results (Group: F = 38.51, p < 0.001; Time: f = 16.57; Interaction: F = 5.92, p < 0.001). At each postoperative time point assessed, NRS scores in the N+TAPB group were significantly lower than those in the C group and the TAPB group (p < 0.05), while NRS scores in the TAPB group were also significantly lower than those in the C group (p < 0.05), demonstrating a stepwise improvement in analgesia across the three regimens. Consistently, BCS scores in the N+TAPB group were significantly higher than those in the C group and the TAPB group (p < 0.05), and BCS scores in the TAPB group remained significantly higher than those in the C group (p < 0.05), indicating superior postoperative comfort with the combined approach. Regarding sedation, Ramsay scores in all three groups remained within 2–3 at each postoperative assessment (calm and cooperative, responsive to commands), and no significant differences were identified between groups (p > 0.05), suggesting that none of the interventions resulted in excessive postoperative sedation. The detailed results are shown in Table 2 and Fig. 2.

**Table 2:** Comparison of postoperative analgesia and comfort scores among the three groups ($\bar{x}$±s).

Index	Time	C group (n = 60)	TAPB group (n = 60)	N+TAPB group (n = 60)	F/U value	p-value
NRS Score	2h	5.83±1.26	4.05±0.79^a^	2.45±0.50^a^^,^^b^	208.318	<0.001
	8h	4.88±1.54	3.68±0.85^a^	1.93±0.73^a^^,^^b^	108.762	<0.001
	12h	4.38±1.66	3.27±1.07^a^	1.55±0.50^a^^,^^b^	88.379	<0.001
	24h	3.80±1.73	2.72±1.19^a^	1.18±0.39^a^^,^^b^	67.800	<0.001
BCS Score	2h	1.43±0.50	2.45±0.50^a^	3.52±0.50^a^^,^^b^	258.616	<0.001
	8h	1.58±0.50	2.53±0.50^a^	3.63±0.49^a^^,^^b^	257.251	<0.001
	12h	2.10±0.71	3.45±0.50^a^	3.78±0.42^a^^,^^b^	155.015	<0.001
	24h	2.12±0.64	3.53±0.50^a^	3.95±0.22^a^^,^^b^	233.727	<0.001
Ramsay Score	2h	2 (1,3)	2 (1,3)	2 (1,4)	0.861	0.424
	8h	2 (1,3)	2 (1,3)	2 (1,3)	0.022	0.978
	12h	2 (1,3)	2 (1,3)	2 (1,4)	1.398	0.250
	24h	2 (1,3)	2 (1,3)	2 (1,3)	1.706	0.185

Note: Compared with Group C.

a p < 0.05.

b Compared with the TAPB group, p < 0.05, 2 h, 8 h, 12 h, and 24 h were the postoperative time points of 2 h, 8 h, 12 h, and 24 h.

### Comparison of hemodynamic indexes among the study groups

Immediately after tracheal intubation, skin incision, and extubation, both MAP and HR in Group C were significantly higher than those in the N+TAPB group and the TAPB group (p < 0.05). Comparatively, no significant differences in MAP or HR were observed between the N+TAPB group and the TAPB group at any of these time points (p > 0.05) (Table 3). To account for potentially differing baseline (T0) values, analysis of covariance (ANCOVA) was performed at T1, T2, and T3 for both MAP and HR using the corresponding baseline value as the covariate. After adjustment for baseline, the group differences remained statistically significant (all p < 0.001 for the omnibus group effect at each of T1, T2, and T3 for both MAP and HR), and the direction and magnitude of the between-group differences were essentially unchanged from the unadjusted analyses shown in Table 3.

**Table 3:** Comparison of hemodynamics at key time points among three groups of patients ($\bar{x}$±s).

Index	Time	C group (n = 60)	TAPB group (n = 60)	N+TAPB group (n = 60)	F value	p-value
MAP (mmHg)	T0	98.42±8.36	97.85±9.12	98.01±8.75	0.258	0.892
	T1	116.12±8.84	102.12±9.40^a^	101.13±8.42^a^	53.243	<0.001
	T2	123.23±8.61	105.13±9.42^a^	104.10±8.51^a^	88.539	<0.001
	T3	119.22±8.90	102.02±9.63^a^	101.10±8.43^a^	77.128	<0.001
HR (times/minute)	T0	82.34±7.89	81.56±8.12	82.01±7.95	0.334	0.756
	T1	98.50±9.48	85.23±9.15^a^	84.80±8.85^a^	43.315	<0.001
	T2	105.33±9.52	88.27±9.23^a^	87.83±9.11^a^	69.294	<0.001
	T3	101.25±9.27	86.03±9.39a	85.72±8.95^a^	55.786	<0.001

Note: Compared with Group C.

^#^Compared with the TAPB group, p < 0.05, T0: Indicates before induction, T1: Indicates immediate tracheal intubation, T2: Indicates immediate skin incision, and T3: Indicates immediate extubation.

a p < 0.05.

### Comparison of postoperative analgesia indicators among the three groups of patients

Within 24 h after surgery, the effective number of PCIA presses in the N+TAPB group was significantly lower than that in the C group and the TAPB group (p < 0.05), and the TAPB group also demonstrated fewer effective presses than the C group (p < 0.05). Similarly, within 24 h postoperatively, the total amount of rescue analgesics used in the N+TAPB group was significantly lower than that in the C and TAPB groups (p < 0.05), while the TAPB group required significantly less rescue medication than the C group (p < 0.05) (Table 4).

**Table 4:** Comparison of postoperative analgesia indicators among three groups of patients ($\bar{x}$±s).

Index	C group (n = 60)	TAPB group (n = 60)	N+TAPB group (n = 60)	F value	p-value
Effective number of presses	18.52±4.46	12.15±3.79^a^	7.82±2.64^a^^,^^b^	126.513	<0.001
Dosage of Paracetamol (mg)	32.50±7.20	22.10±6.50^a^	12.50±4.80^a^^,^^b^	98.765	<0.001
Flurbiprofen dosage (mg)	11.73±3.50	5.92±2.20^a^	3.30±1.50^a^^,^^b^	85.432	<0.001

Note: Compared with Group C.

a p < 0.05.

b Compared with the TAPB group, p < 0.05. PCIA, patient-controlled intravenous analgesia.

### Comparison of postoperative recovery quality and complications among three groups of patients

At 24 h after surgery, the QoR-15 score in the N+TAPB group was significantly higher than that in the C group and the TAPB group (p < 0.05), and the TAPB group also showed a significantly higher QoR-15 score than the C group (p < 0.05). The mean QoR-15 score of 128.7 in the N+TAPB group versus 92.9 in the C group corresponds to a between-group difference of approximately 35.8 points, which is substantially larger than the reported Minimal Clinically Important Difference (MCID) for QoR-15 of approximately 6–8 points,^17^ supporting both the statistical and clinical relevance of this finding. With respect to postoperative adverse events, the incidences of nausea and vomiting, as well as pruritus, were significantly lower in the N+TAPB group than in the C group (p < 0.05) (Table 5). For the omnibus χ^2^ test of nausea and vomiting incidence across the three groups, the result was borderline (χ^2^ = 6.000, exact p = 0.050). Pairwise comparisons using Fisher’s exact test with Bonferroni correction (adjusted α = 0.017 for three pairwise tests) showed that the difference between Group C (25.0%) and Group N+TAPB (8.3%) reached the corrected significance threshold (p = 0.015, adjusted), whereas the comparisons C vs. TAPB (25.0%vs. 16.7%) and TAPB vs. N+TAPB (16.7%vs. 8.3%) did not (both adjusted p > 0.05). Findings for pruritus followed the same direction.

**Table 5:** Comparison of postoperative 24-h QOR-15 scores and incidence of complications among three groups of patients.

Index	C group (n = 60)	TAPB group (n = 60)	N+TAPB group (n = 60)	Statistical value	p-value
Nausea and vomiting (n)	25.0 (15/60)	16.7 (10/60)	8.3 (5/60)^a^	χ^2^ = 6.000	0.050
Itchy skin (n)	13.3 (8/60)	10.0 (6/60)	3.3 (2/60)^a^	χ^2^ = 3.841	0.146
Dizziness (n)	8.3 (5/60)	6.7 (4/60)	5.0 (3/60)	χ^2^ = 0.536	0.765
Respiratory depression (n)	3.3 (2/60)	1.7 (1/60)	0 (0/60)	χ^2^ = 2.034	0.362
QOR-15 rating (points, *χ*±s)	92.93±11.15	110.07±15.34^a^	128.73±11.81^a^^,^^b^	F = 115.633	<0.001

Note: Compared with Group C.

a p < 0.05.

b Compared with the TAPB group, p < 0.05. QoR-15, Quality of Recovery-15 scale.

## Discussion

Preemptive analgesia and multimodal analgesia are well-established strategies in perioperative pain management, and accumulating evidence indicates that preemptive analgesia can improve postoperative pain control and reduce analgesic requirements.^18^ The Expert Consensus on Perioperative Goal-Oriented Pain Management^19^ emphasizes preemptive analgesia as an integral component of perioperative pain management and recommends adopting multimodal analgesia to enhance analgesic efficacy and safety. In line with this concept, Kim et al.^20^ incorporated multimodal principles into preemptive regimens and proposed “multimodal preemptive analgesia”, reporting improved postoperative pain control in lumbar surgery.

In the present study, the N+TAPB regimen provided better postoperative analgesia than either nalbuphine or subcostal TAPB alone, and the between-group reduction in NRS scores exceeded the commonly reported Minimal Clinically Important Difference (MCID) of approximately 2 points for acute postoperative pain, supporting not only statistical but also clinical relevance. Nalbuphine is primarily a κ-opioid receptor agonist with partial μ-opioid receptor antagonistic/agonistic properties, and it may be particularly effective for visceral pain and diaphragmatic referred pain, while potentially reducing μ-receptor-mediated adverse effects such as respiratory depression, nausea, and vomiting.^21^^,^^22^ In terms of mechanism, activation of the κ-receptor can inhibit the nociceptive information transmission in the spinal dorsal horn and down regulate the excitability of visceral afferent nerves, thereby synergistically playing an anti-nociceptive effect at the spinal cord and peripheral levels. In this study, nalbuphine 0.1 mg/kg was injected intravenously 15-min before anesthesia induction, which is in line with the core idea of preemptive analgesia, that is, to establish the basis of central analgesia before the introduction of noxious stimulation, so as to limit the formation of central sensitization and improve the postoperative analgesic outcome.^23^ Subcostal TAPB, performed under ultrasound guidance within the transversus abdominis plane, can block the anterior and lateral cutaneous branches of the lower thoracic intercostal nerves (T7–T12), providing coverage of the upper abdominal wall relevant to trocar insertion and incision pain in LLR.^24^ Since pain after LLR is typically mixed, involving both abdominal wall somatic pain and a visceral component,^25^ combining nalbuphine with subcostal TAPB offers complementary analgesic mechanisms that more comprehensively address postoperative nociception. In addition, although opioids remain important components of postoperative analgesia, higher opioid exposure is associated with a greater incidence of adverse effects, including nausea, vomiting, and pruritus.^26^ Consistent with this principle, patients in the N+TAPB group required fewer additional analgesics and experienced lower rates of nausea, vomiting, and pruritus than those in the control group, which is broadly consistent with prior reports of multimodal regimens reducing opioid-related adverse events.^27^

The results of this study indicate that the combined regimen was associated with clear advantages in hemodynamic stability, improved early postoperative recovery quality, and a lower incidence of complications. Surgical stimulation activates the sympathetic nervous system, which can cause marked increases and fluctuations in HR and MAP, thereby increasing the risk of cardiovascular and cerebrovascular events.^28^ Although hemodynamic parameters did not differ significantly between the TAPB and N+TAPB groups, the superiority of the combined regimen was primarily reflected in improved analgesic efficacy, reduced rescue analgesic consumption, and higher QoR-15 scores, rather than additional hemodynamic benefit. In Group C, neither preemptive analgesia nor regional blockade was used, and nociceptive input was therefore transmitted more directly to the central nervous system, resulting in a stronger perioperative stress response. In the TAPB group, regional blockade primarily attenuated abdominal wall somatic pain, which likely reduced the overall stress response to some extent, whereas preemptive nalbuphine in the N+TAPB group further suppressed visceral nociception and stress-related responses.^29^ Although the differences in hemodynamic parameters between the TAPB and N+TAPB groups did not reach statistical significance ‒ possibly because TAPB alone already provided substantial stress attenuation or because the study was not powered to detect smaller incremental effects ‒ both groups demonstrated significantly greater hemodynamic stability than the control group, underscoring the importance of adequate analgesia in perioperative hemodynamic control.

Within 24 h after surgery, patients in the N+TAPB group had significantly fewer effective PCIA presses and lower rescue analgesic consumption than those in both the C group and the TAPB group, suggesting that the combined regimen achieved more satisfactory analgesia through complementary mechanisms. Preemptive nalbuphine primarily targets visceral pain and diaphragmatic referred pain, while subcostal TAPB blocks abdominal wall somatic pain; together, these modalities more comprehensively cover the major pain components after LLR and thereby reduce the need for additional analgesia.^30^ In the C group, inadequate pain control due to the absence of both preemptive analgesia and regional blockade was reflected by higher PCIA activation and greater rescue analgesic use. In the TAPB group, analgesia was largely limited to the somatic component, and residual visceral pain may explain the continued requirement for supplemental analgesia. Importantly, the synergistic effect of the combined approach not only strengthened analgesic efficacy but also improved recovery quality by reducing opioid-related adverse events (e.g., nausea, vomiting, and pruritus), which aligns with ERAS principles.^31^

Postoperative analgesia is a cornerstone of ERAS pathways after LLR, and the combined regimen evaluated in this study contributes directly to several ERAS objectives. Effective analgesia facilitates early mobilization and improves respiratory mechanics, which may reduce postoperative complications such as pulmonary infection and venous thromboembolism. In addition, improved hemodynamic stability and attenuation of stress responses may lower the risk of postoperative organ dysfunction, while fewer complications may translate into shorter hospital stays and reduced healthcare costs. Given its relative procedural feasibility and favorable safety profile, this combined approach may also apply to other laparoscopic procedures in hepatobiliary surgery.^32^

This study has several limitations. First, and most importantly, the control group did not receive a sham TAPB procedure. Consequently, the observed differences between Group C and the TAPB groups may include a procedural placebo effect arising from the act of ultrasound positioning, needling, injection, and patient expectation, and the authors cannot isolate the specific pharmacological contribution of the TAPB from these procedural factors. Therefore, the present findings should be interpreted as showing the benefit of the regimen of preemptive nalbuphine plus TAPB compared with no regional block, rather than as a pure mechanistic demonstration of TAPB’s pharmacological efficacy. Second, patients and anesthesiologists were not blinded to the intervention; although outcome assessors were blinded to group assignment, the absence of patient blinding may introduce expectation bias, particularly for the patient-reported NRS, BCS, and QoR-15 scores. Third, no objective measure of block success (e.g., dermatomal sensory testing) was performed, so the technical success of TAPB in every patient cannot be independently confirmed. Fourth, participants were recruited from a single center (Huizhou Central People’s Hospital), which may limit generalizability and introduce regional or practice-related bias; therefore, multi-center studies are needed to validate these findings. Fifth, outcomes were evaluated only within the first 24 h postoperatively, without assessment of longer-term analgesia and recovery at 48 and 72 h, and chronic post-surgical pain was not investigated. Sixth, multiple secondary outcomes (QoR-15, adverse events, hemodynamics) were analyzed without a formal adjustment for multiplicity across the family of secondary endpoints (Bonferroni correction was applied only to post-hoc pairwise comparisons within each outcome), so the risk of false-positive findings for secondary endpoints cannot be entirely excluded. Finally, residual confounding related to technical variability among surgeons cannot be completely excluded. Lastly, the authors did not explore different nalbuphine doses or local anesthetic concentrations, and dose-response relationships remain unclear. In addition, the sample size of this study was calculated by referring to the incidence of moderate and severe pain in the study related to esophagectomy. Although this study is highly comparable with this study in terms of analgesic scheme design and definition of outcome indicators, and it is a high-quality literature available at that time, there are differences between esophagectomy and laparoscopic hepatectomy in terms of surgical trauma scope, incision location and pain source, which may affect the extrapolation accuracy of effect quantity. In this study, the sample size has been adjusted in combination with the pre-test data, but there is still a potential possibility of bias in the estimation of effect size due to the difference in operation type. Future multi-center, adequately powered randomized controlled trials should optimize dosing strategies and local anesthetic concentrations and extend follow-up to evaluate longer-acting and individualized analgesic protocols.

In conclusion, the regimen of preemptive intravenous nalbuphine combined with ultrasound-guided subcostal transversus abdominis plane block was associated with improved postoperative analgesia, more stable perioperative hemodynamics, better recovery quality, and fewer opioid-related adverse events after laparoscopic hepatectomy than no regional block, and it represents a feasible multimodal analgesia strategy in this setting. The combined regimen has clinical value in improving the quality of analgesia after laparoscopic hepatectomy and reducing opioid related adverse reactions. However, since the control group did not receive a sham TAPB procedure, the exact pharmacological contribution of TAPB could not be completely independent of the placebo effect related to the procedure itself. This conclusion should be further verified by future multi-center trials incorporating a more rigorous control design with a sham TAPB control and patient blinding.

## Ethics approval

Ethical approval was obtained from the Ethics Committee of Huizhou Central People's Hospital (Approval n° KYll2024085).

## Consent to participate statement

Written informed consent was obtained from a legally authorized representative(s) for anonymized patient information to be published in this article.

## Authors’ contributions

Fang He: Designed the study and carried them out. Fang He, Peng Jiang, Dexing Luo: Supervised the data collection. Fang He, Peng Jiang, Dexing Luo: Analyzed the data. Fang He, Peng Jiang: Interpreted the data. Fang He, Dexing Luo: Prepared the manuscript for publication and reviewed the draft of the manuscript. All authors have read and approved the manuscript.

## Funding

This work was supported by Science and Technology Planning Project of Huizhou City, Guangdong Province (Grant n° 2024CZ010285).

## Declaration of competing interest

The authors declare no conflicts of interest.

## Data Availability

The data that support the findings of this study are available from the corresponding author upon reasonable request.
